# Study on the influencing factors of childcare needs and their operation mechanism among parents with children aged 0–3 years in China—an empirical analysis based on a mixed methods study

**DOI:** 10.3389/fpubh.2025.1353947

**Published:** 2025-06-09

**Authors:** Huanhuan Li, Bingyu Duan, Yuhuan Zhang, Mingci Chen, Yifang Wang, Huijuan Di

**Affiliations:** ^1^College of Educational Science, Xinjiang Normal University, Urumqi, China; ^2^School of Education Science, Hanshan Normal University, Chaozhou, China; ^3^Shanghai Institute of Early Childhood Education, Shanghai Normal University, Shanghai, China; ^4^Department of Preschool Education, Hebei Normal University, Shijiazhuang, China

**Keywords:** Grounded Theory, childcare, childcare needs, influencing factors, mixed method

## Abstract

**Background:**

Exploring and analyzing the factors influencing parents’ demand for childcare is essential for improving the quality of childcare services. However, there is a lack of empirical studies that effectively explore the factors influencing demand for childcare and its mechanisms of action. Therefore, this study used a mixed research method to explore the factors influencing demand for childcare and their mechanisms of action.

**Methods:**

Semi-structured interviews were conducted with 63 participants including parents of 0–3-year-old children, childcare center administrators, teachers, education specialists, and community members. Second, three levels of coding of the interview data using Grounded Theory were used to summarize the factors influencing parents’ childcare needs. Third, we combined relevant theories to construct a theoretical model of the factors influencing parents’ childcare services for children aged 0–3 years old and formulated research hypotheses. Fourth, the study used the “Questionnaire on the Childcare Needs of Parents of Children Aged 0–3″ to survey 516 parents. We analyzed and tested the constructed theoretical model through regression and mediation analyses.

**Results:**

The Grounded Theory results found that factors influencing the childcare needs of parents of infants and toddlers aged 0–3 years include Childcare Center Management, Teacher Professionalism, Environment and Facilities, Health Care Services, and Educational Activities. Mediating effect analyses revealed that (1) Childcare Center Management and Teacher Professionalism positively and directly influenced Educational Activities. (2) Health Care Services is mediated between Childcare Center Management and Educational Activities, and Health Care Services is mediated between Teacher Professionalism and Educational Activities. (3) Environment and Facilities and Health Care Services are chained between Childcare Center Management and Educational Activities, Teacher Professionalism and Educational Activities.

**Conclusion:**

Childcare-related organizations should improve the quality of Childcare Center Management, Teacher Professionalism, Environment and Facilities, Health Care Services, and Educational Activities to eliminate parents’ childcare concerns and meet their childcare needs.

## 1 Introduction

Numerous studies have shown that the first 3 years of a child’s life are the prime time for brain development (1–4). The life experiences and educational experiences gained by infants and toddlers during this period influence the individual’s ability to form and develop perceptions, memory, thinking, and personality (5, 6). Therefore, the care and upbringing of infants and toddlers require extremely high quality environmental conditions to support their development. The family is essential for infants and young children’s physical and mental development as the primary environment that supports their survival and development (7). However, with societal development, families face multiple pressures from work, the economy, and family conflict in caring for infants and young children. For example, working women’s difficulties in combining employment and childcare, lack of funds and skills, parents’ lack of sleep, and strained family relationships (8). When there is a lack of necessary support in long-term care, prolonged stress can lead to mental health problems for parents of infants and toddlers, causing them to become more irritable and impatient in the care of their infants and toddlers. This negative caregiving style may interfere with forming healthy attachments and lead to various behavioral problems in infants and toddlers (9, 10). Childcare services refer to social service mechanisms that supplement the family’s childcare function when, for different reasons, the childcare function within the family is not functioning correctly (11). Published research has shown that childcare positively impacts the development of infants and toddlers and their families. On the one hand, high-quality childcare services can promote the cognitive, linguistic, behavioral, and socio-emotional development of infants and toddlers, alleviate inequalities in early childhood development, and effectively prevent unintentional injuries, illnesses, and even deaths (12–14). On the other hand, childcare services can reduce the burden of raising and educating families, facilitate women’s employment development and the realization of work-family balance, enhance the quality of life of families, and contribute to the happiness and harmony of families with infants and young children (15–17).

In recent years, China’s ongoing adjustments to its birth policies have placed increasing pressure on family caregiving. In particular, after implementing the “comprehensive two-child” policy, Chinese families have had a growing demand for childcare services (18). Parents’ need for childcare has expanded from primary life care to more demanding aspects such as educational guidance for infants and toddlers (19). Chinese governments hope to solve or alleviate the problems of family childcare by establishing a sound system of childcare services, providing social support and social welfare, and compensating for the shortcomings of parental care, protection, and education (20). In May 2019, the General Office of the State Council issued the Guiding Opinions on Promoting the Development of Care Services for Infants and Toddlers under three Years of Age (21), which, for the first time, emphasized the importance of infant and toddler care and clarified governmental responsibilities. This initiative indicates that infant and toddler care services will soon be fully integrated into the national public services system (22). Subsequently, the Ministry of Education, Health Commission, State Council, and other relevant departments have issued a series of policies, making detailed provisions on setting standards for childcare institutions, institutional management norms, education, and financial support, accelerating the development of China’s childcare service system. According to statistics, the number of infants and toddlers aged 0–3 years old in China is approximately 32 million, of which more than 30% of families have expressed a need for childcare. However, the enrollment rate of infants and toddlers in China is only 6% (23). It can be seen that China’s existing childcare service system is not yet perfect, and the supply imbalance is apparent (24).

Some childcare centers may focus too much on economic benefits in marketing, which to some extent has caused a series of problems: imbalance in the supply structure, lack of public childcare institutions, lack of care institutions providing both infant care and education services, insufficient supply of care services, low level of teachers, insufficient health care, and lack of supervision and management of the quality of institutions (25–27). Many scholars have studied the factors influencing parents’ demands for childcare to solve the above problems and promote childcare services’ development. Existing studies have focused on two main areas: (1) analyzing the relationship between family background and parents’ demand for childcare services. (2) Exploring the impact of childcare service quality on parents’ demand for childcare services. On the family background, some scholars have found that parents’ educational background, age, occupation, income, family location, and family structure influence parents’ childcare demands (28–33). For example, parents with higher educational levels are more likely to invest money in childcare services for their children’s development (28, 29). Urban women face more severe work-child conflicts, and urban mothers are more willing to pay for childcare than rural mothers (34). In addition, there are differences in the demand for infant and toddler childcare services among families with different numbers of children: the greater the number of children, the greater the need for childcare providers’ help (35).

Some scholars have also explored the impact of factors such as the price and quality of childcare services and the geographic location of childcare facilities on the demand for childcare services in terms of childcare facility factors (35, 36). For example, Xu pointed out that when choosing a childcare institution, parents of infants and toddlers pay the most attention to the qualifications and competence of the staff, followed by the quality of the educational program, the quality of the childcare environment, and the condition of the facilities, and put the geographical location and proximity of the distance to a relatively secondary position (37). Subsequently, He et al. pointed out that parents of infants and young children most valued home care distance and teacher competence, among others, when choosing childcare providers (38). Bssok et al. found that parents strongly preferred caregivers’ educational attainment in terms of the quality of childcare (39).

Regarding research content, existing studies have mainly focused on the influence of family background characteristics on parents’ demands for childcare. Although a few scholars have paid attention to the relationship between the quality of childcare institutions and parents’ demands for childcare, they have not reached a unanimous conclusion on the matter. At the same time, there is no unified conclusion or exploration of the relationship between childcare institution factors. In terms of research methodology, past studies have often used a single quantitative method (34, 37, 38), such as questionnaires and experimental methods. There is a lack of exploration of the factors influencing childcare needs using qualitative methods such as interviews, Grounded Theory, and mixed research methods. Therefore, this study first used the interview method and Grounded Theory (GT) to explore the factors of childcare providers that influence parents’ childcare needs. Second, the relationships between childcare provider factors were analyzed to construct a theoretical model of the factors influencing parental childcare demand. Finally, the questionnaire method was used to collect empirical data to validate our theoretical model and provide academic support and a practical reference for the high-quality development of childcare services. The following two research questions guided our study:

(1) Which factors influence parents’ childcare needs?
(2) What are the mechanisms of action of these factors?

## 2 Research design

**Figure 1:**
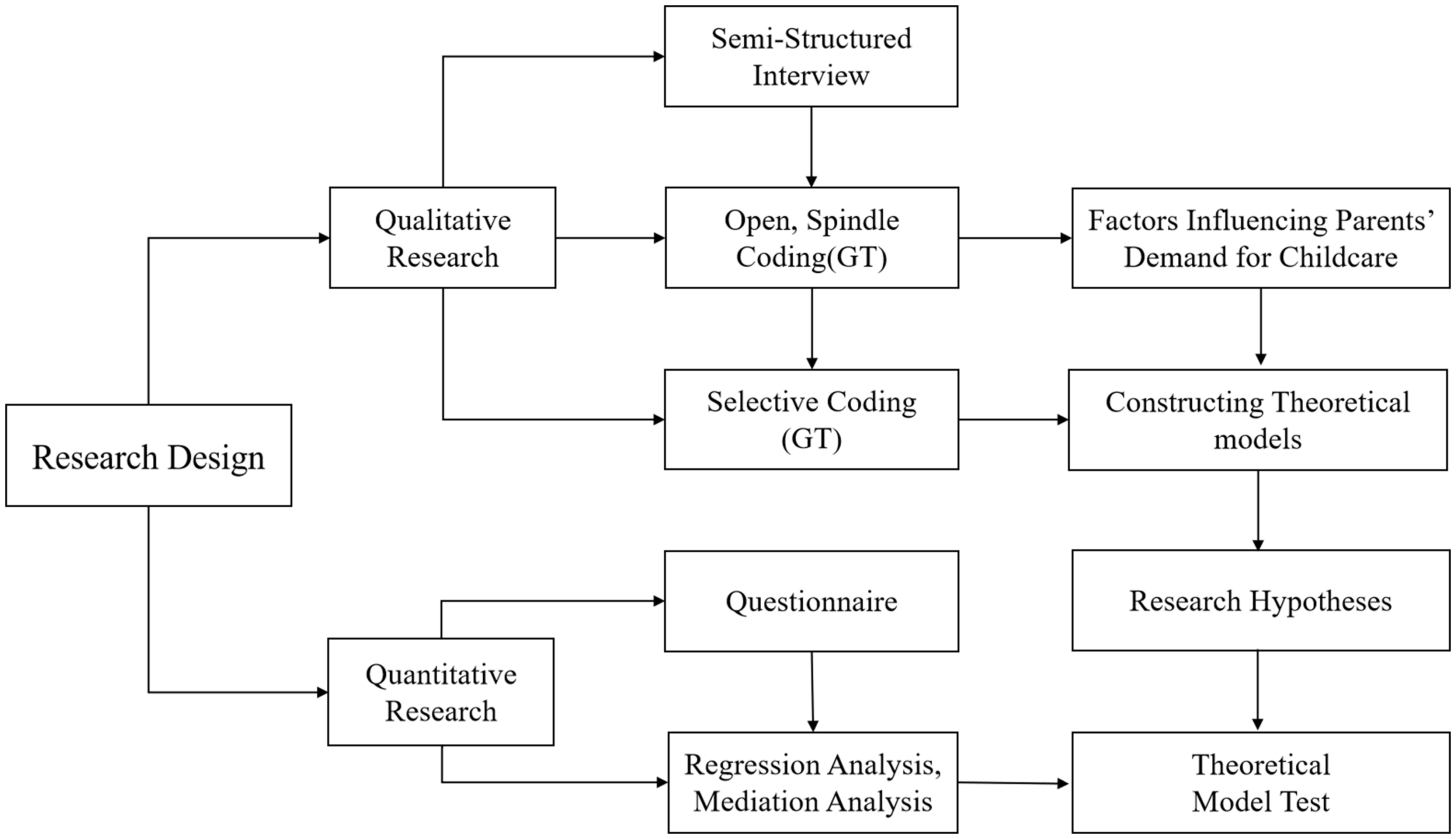
Research step.

This study used a mixed method to explore the influencing factors and their operation mechanisms for the children’s childcare needs of 0–3-year-old children’s parents. First, parents of children aged 0–3 years, childcare center managers, childcare teachers, education specialists and community members were selected, totalling 63 individuals. Semi-structured interviews were conducted with them to generate interview data. Second, the qualitative data obtained from the interviews were coded at three levels using GT, summarizing the factors influencing the childcare needs of children aged 0–3 years and combining relevant theories for theoretical modeling. Third, the study was based on the constructed theoretical model with corresponding research hypotheses, and the questionnaire was used to obtain quantitative data combined with regression analysis and mediating role analysis to test the research hypotheses. The study design is illustrated in Figure 1.

## 3 Qualitative research design and the theoretical model

### 3.1 Grounded Theory and interview

We used a GT approach to establish the factors and mechanisms influencing the childcare needs of parents of children aged 0–3 years. Purposive sampling and snowball sampling were used for sample selection. According to the principle of purposive sampling, the interviewees needed to fulfill the following conditions:

- Have a preliminary understanding of childcare for 0–3-year-olds.
- Have the willingness and experience of childcare or childcare staff.
- Understand the interview questions and answer their personal questions.
- The entire interview process was recorded.

A small number of sample subjects were first sought through purposive sampling. Then, snowball sampling was used to finalize the 63 interviewees, including parents with children aged 0–3 years, administrators of childcare facilities, childcare teachers, education experts, and community workers. Unstructured interviews were used to obtain information for GT to establish the theory, and unstructured one-to-one telephone, WeChat, and face-to-face interviews were conducted. Every interview took 30–60 min, and each interviewee’s information was treated as confidential. After obtaining the interviewees’ consent, we recorded and collated the interview elements. Demographic statistics of the interview samples are listed in Table 1.

**Table 1:** Summary of demographic characteristics of the interview sample.

Item	Type	Frequency	Percentage (%)
Genders	Male	19	30.2
	Female	44	69.8
Education Background	Middle school	4	6.3
	High school	10	15.9
	College	26	41.3
	Bachelor’s degree	19	30.2
	Graduate degree	4	6.3
Interview Type	Parents with children aged 0–3	22	34.9
	Childcare institution managers	12	19.0
	Childcare teachers	18	28.6
	Education experts	7	11.1
	Community members	4	6.3
Total		63	100

### 3.2 Coding analysis

After identifying the research question on childcare needs and collecting and analyzing the primary data (in Chinese), the primary data were coded at three levels (40) to develop a framework and dimensions for interpreting childcare needs. To maximize the objectivity of the coding results, two researchers did the coding process independently. They used Nvivo12 software to organize and analyze the raw textual information sentence by sentence. The initial concepts were also categorized and clustered based on similarity, causality, and other relationships. Subsequently, with the help of the code comparison function in the Nvivo12 software, a code consistency test was carried out, whereby coded content with a kappa coefficient of less than 0.75 was repeatedly discussed, and concepts with a low conceptual repetition rate or a high degree of similarity were eliminated, refined, or merged by consulting with experts until the consistency ratio was 85% or more, with the specific coding steps being as follows.

#### 3.2.1 Open coding

This is a preliminary step in analyzing data that involves scrutinizing, comparing, and conceptualizing data to identify potential concepts, categories, and attributes. Each word, sentence, and paragraph about childcare needs in the raw interview data was interpreted and labeled semantically to form the initial concepts. These initial concepts were then summarized, clustered, and analyzed to form 49 initial concepts, as shown in Table 2.

**Table 2:** Example of open coding process.

Initial concept	Label	Original sentence
Nutrition and Dietary Management	Nutrition monitoring, Feedback	I expect the childcare provider to check my child’s nutritional status regularly. If there is a problem, I expect them to inform me so we can make timely adjustments. (^a^P)
	Nutritional balance, Dietary diversity	Parents are very concerned that their children are eating a balanced diet, and they expect us to provide a variety of foods to ensure that their children are getting the necessary nutrients. (^C^T)
	Meal arrangements, Meal times	Many parents ask us about their children’s eating arrangements, and they want their children to have regular meal times and healthy eating habits. (^b^A)
Toys, Teaching Aids and Materials	Exploration, Creativity, Toys, Teaching Aids, Materials	Parents expect childcare providers to provide various toys, teaching aids, and materials to promote their children’s exploration and creativity. (^C^T)
	Safety, Non-Toxicity	I want child care providers to provide safe, non-toxic toys to ensure children’s health. (^a^P)
	Materials, Development needs	As professionals, we must ensure that the materials and facilities meet the developmental needs of children at different ages. (^d^E)
Outdoor Activities and Sunshine	Fresh air, Sunlight, Outdoor activities	It’s crucial for children’s physical and mental development to have regular access to fresh air and sunlight. These natural elements are essential for their overall well-being and growth.” (^e^C)
	Sunlight, Bone development	Sunlight is vital for bone development and vitamin D synthesis; parents want their children to have enough sunlight in their childcare facilities. (^d^E)
	Outdoor activities	We have learned that many parents value their children’s outdoor time and want childcare providers to offer abundant outdoor activities. (^b^A)

a P = result of parent interview.

b A = result of administrator interview.

C T = result of teacher interview.

d E = result of experts interview.

^eC = result of community workers interview.^

#### 3.2.2 Axial coding

This is another crucial step in rooted theory. Axial Coding aims to integrate dispersed concepts and discover their causal relationships, interactions, conditions, and consequences. Principal axis coding helps to incorporate the concepts generated by open coding and discover the complex relationships among them, thus constructing a finer and deeper organic whole. In this paper, 16 subcategories were obtained through principal axis coding, and five main categories were summarized, as shown in Table 3.

**Table 3:** Categories and their attributes and dimensions.

Main category	Sub-category	Concept
Childcare Center Management (CCM)	Healthcare work Management	Health Surveillance and Nursing
		Nutrition and Dietary Management
		Sudden Illness and Accident Management
	Educational and Teaching work	Education Programs and Implementation
		Educational Resource Allocation
		Infant and Toddler Development Assessment and Feedback
	Logistics and Safety Management	Safety Regulations and Training
		Facility and Environmental Safety Maintenance
		Emergency Response and Incident Management
Teacher Professionalism (TP)	Professional Ethics and Morality	Infant and Toddler Care Philosophy
		Professional Ethics and Morals
		Teacher Self-growth and Reflection
	Professional Knowledge	Infant and Toddler Developmental Psychology
		Early Education Theory
		Family and Social Cooperation
	Professional Skills	Infant and Toddler Care Skills
		Instructional Design and Innovation
		Parent Communication and Cooperation
Environment and Facilities (EF)	Material Facilities	Activity Space Design and Layout
		Toys, Teaching Aids and Materials
		Environment, Facilities and Renewal
	Spiritual Culture	Culture and Values
		Mental Health and Emotional Development of Infants and Toddlers
		Facilities-Home Cooperation and Social Support
Health Care Services (HCS)	Physical Exercise	Daily Physical Activity
		Outdoor Activities and Sunshine
		Safety Guidelines for Physical Activity
	Diet Management	Food Safety and Hygiene Management
		Nutritionally Balanced Meals for Infants and Toddlers
		Eating Behavior and Habit Development
	Disease Prevention and Control	Prevention and Management of Infectious Diseases
		Cultivation of Daily Hygiene Habits
		Health Education and Promotion
	Health Management	Regular Health Screening and Assessment
		Infant and Child Health Record Management
		Individualized Health Management Plan
	Injury Prevention	Safety Education for Infants and Toddlers
		Inspection and Maintenance of Safety Facilities
		Emergency Plan and Handling of Accidents
Educational Activities (EA)	Life-style Education Activities	Development of Daily Living Skills
		Self-Management and Independence
		Common Sense and Rules Education
	Play-based Educational Activities	Support of Play Environment and Materials
		Guidance and Interaction in Play Activities
		Cultivation of Children’s Socialization and Cooperation Skills
	Inquiry-based Educational Activities	Exploration and Curiosity Development
		Scientific Inquiry and Problem Solving
		Creative Thinking and Practice
		Assessment and Feedback on Learning Outcomes for Infants and Toddlers

#### 3.2.3 Selective coding

This is a further streamlining and condensation of the main categories, and the core categories are explored and used as the “story line” to construct the theoretical framework, systematically explain the association between the categories, and form a typical relationship structure between the main categories. In this study, we analyzed the five main categories formed by the axial coding, explored the relationship between the main categories and the childcare needs of parents of infants and toddlers, and found that the interactions between the main categories would also affect the childcare needs of parents of infants and toddlers, as shown in Table 4.

**Table 4:** Relationship between selective encoding paths.

Index	Cause path	Structural relationship	Internal meaning
1	TP-EA	Direct impact	TP has a direct effect on EA
2	CCM-EA	Direct impact	CCM has a direct effect on EA
3	TP-HCS-EA	Mediation	TP influences EA through HCS
4	TP-EF-HCS-EA	Chain mediation	TP influences HCS through the EF, which in turn has an impact on EA
5	CCM-HCS-EA	Mediation	CIM influences EA through HCS
6	CCM-EF-HCS-EA	Chain mediation	CCM influences HCS through the EF, which in turn has an impact on EA

### 3.3 Testing of theory saturation

We used the remaining 13 randomized data from the interviews as a saturation test sample to test the theoretical saturation of the findings. No new conceptual categories or relationships were identified during the testing. This indicates that the original GT fully accommodated the relevant categories and concepts, implying that the model passed the theory saturation test.

### 3.4 Construction of theoretical model

The coding and analysis of the interview data led to identifying five core categories: Childcare Center Management (CCM), Teacher Professionalism (TP), Environment and Facilities (EF), Health Care Services (HCS), and Educational Activities (EA). CCM refers to managing healthcare, educational teaching, and logistics safety in daycare institutions using two different models: the refined standardized model and the rough and chaotic model. TP refers to the various levels of professional knowledge, skills, and qualities of early childhood education educators and whether they have obtained relevant professional certificates. EF refers to the material facilities and cultural atmosphere of childcare centers in two different spaces, internal and external, with other qualities and utilization levels. HCS relates to the services provided by childcare institutions in various forms, such as full-day, half-day, and temporary care, to give the children different qualities of physical exercise, dietary management, disease prevention and control, health management, and injury prevention. EA refers to other attributes of life, games, and teaching activities provided by childcare institutions through child-game-based, teacher-led, and home-based cooperation.

We constructed a model with six paths based on the interview data, existing theory, and relationships among the five core categories. As shown in Table 4, the professional qualifications of teachers in these categories were directly related to EA, HCS, and EF. The management of childcare institutions is directly related to EA, EF, and HCS. EF is directly associated with HCS, whereas HCS is associated with EA. Simultaneously, the path from EF to HCS played a mediating role in teachers’ professional qualifications.

**Figure 2:**
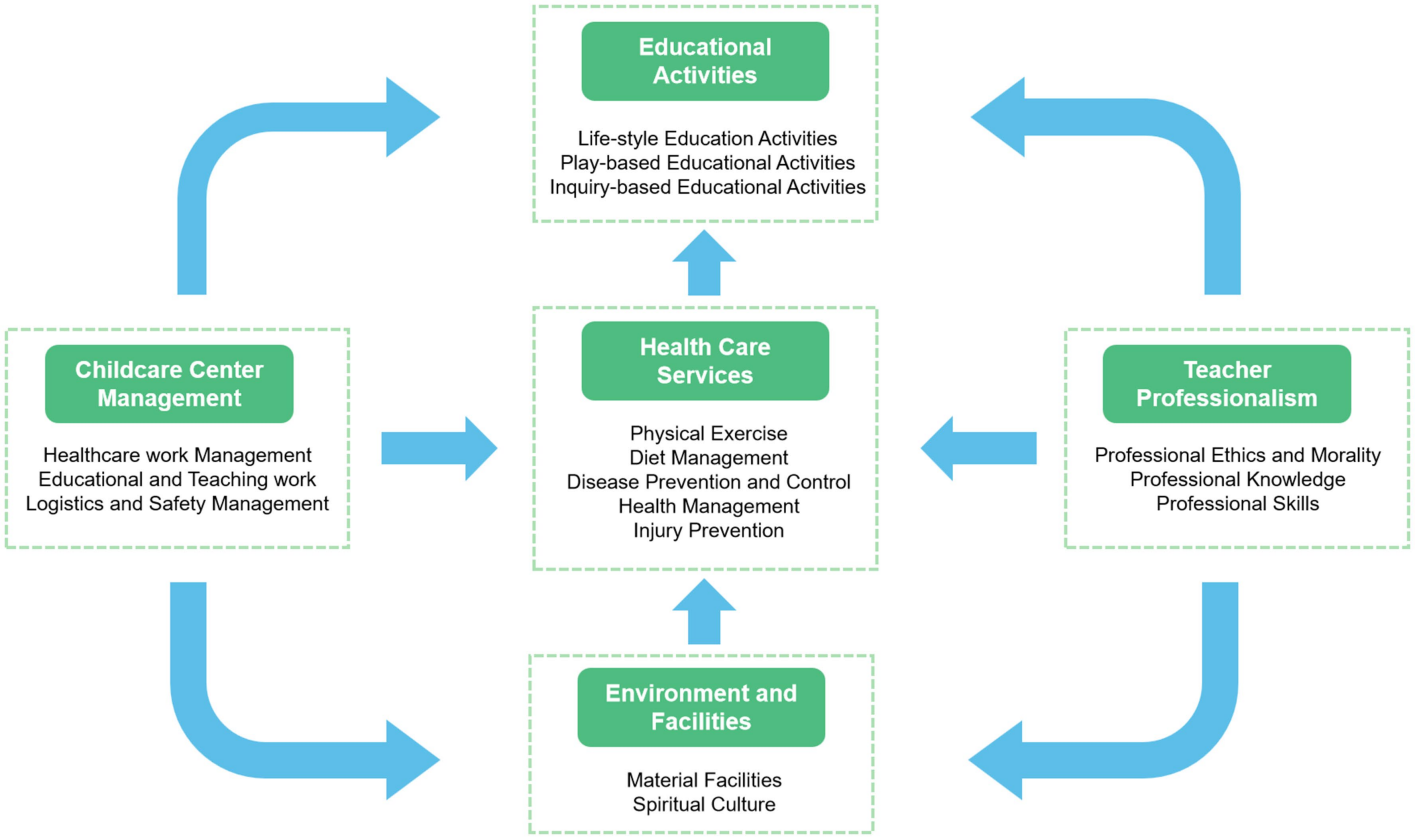
Theoretical model.

Similarly, the professional qualifications of teachers indirectly affect EA through HCS, and CCM indirectly affects HCS through EF and EA through HCS. In addition, the path from teachers’ professional qualifications to EA was also affected by the common chain-mediated effects of EF and HCS. Similarly, CCM affects HCS by influencing EF and indirectly affects EA. Finally, based on the third level of coding analysis, a model diagram of the core category “Factors Influencing the Demand for Childcare and Their Mechanism of Action” was constructed (as shown in Figure 2).

**Paths one: CCM→CS**. Security and routine management of teaching and learning are essential guarantees for the regular operation of education and teaching in educational institutions and the overall quality of academic activities (41). CCM directly affects the quality of EF as a critical safeguard for maintaining its daily work.

**Paths two: TP→EA**. Professionalism among early childhood teachers is a vital and central factor in implementing educational activities. This directly affects the quality and effectiveness of these activities (42). In young children’s behavior, Barandiaran et al. found that teachers’ sensitivity was positively related to young children’s exploratory behavior: the more significant the teacher’s sensitivity, the more exploratory behaviors exhibited by the children in care (43). Howes et al. pointed out that when children experience higher-quality teaching or tighter teacher-student relationships, they significantly impact academic performance (44). Aguillard et al. suggested that caregivers’ caregiving capacity is vital for implementing continuity of care. When caregivers receive more positive feedback, young children’s cognitive ability is enhanced. The communication skills of young children are reinforced (45). If TP is not high, it will hinder the improvement of the quality of educational activities in childcare institutions.

**Paths three: TP→HCS→EA**. Education and childcare are interpenetrating, interlinked, and interactive in early childhood. Zhong suggested that childcare is a direct pathway for teachers to become deeply involved and intervene in the routine lives of children. HCS quality affects EA quality and the degree to which the goal of young children’s whole and harmonious development is achieved (46). From the perspective of childcare teachers, Tao found that the development and science management of the childcare teacher workforce affects HCS quality (47). Similarly, Shen et al. found a lack of specialized early childhood healthcare providers constraining HCS and young children’s healthy physical and mental development (48).

**Paths four: CCM→HCS→EA**. Previous studies have shown that CCM influences EA through the HCS. For example, Zhao believed detailed and standardized management improved work efficiency and management levels and enhanced childcare quality and hygiene (49). Using quantitative methods, Biersteker et al. assessed the quality of childcare and child education and found that childcare centers’ managerial abilities predicted care levels (50).

**Paths five: TP→EF→HCS→EA**. First, teachers are the most critical factor influencing the quality of the childcare center’s environment, and they are an essential component of the structural quality of the childcare center’s environment (51). Hong et al. also point to the lack of talented teachers as an essential obstacle to guaranteeing and promoting the quality of young children’s environments (52). Second, a facility’s physical environment is one of the main elements of childcare structure quality (53). Tonge et al. found that outdoor environments, facilities, and size influence children’s physical activity. Larger outdoor play spaces provide more opportunities for children to move freely and increase their physical activity (54). Therefore, TP influences HCS through EF, which in turn impacts EA.

**Paths six: CCM→EF→HCS→EA**. Studies have shown that CCM influences EA through EF and HCS. For example, Adamo et al. studied unlicensed day care centers (UDC). They found that a lack of management led to green spaces lacking diversity, quality, and quantity and harmed the HCS (55). Rupprecht et al. study physical activity interventions for basic motor skills in preschoolers in childcare centers. This study showed that childcare centers that provide a stimulating and supportive environment can promote the development of young children’s motor skill potential, increasing the effectiveness of EA (56).

## 4 Quantitative research design and data analysis

### 4.1 Research hypotheses

**Figure 3:**
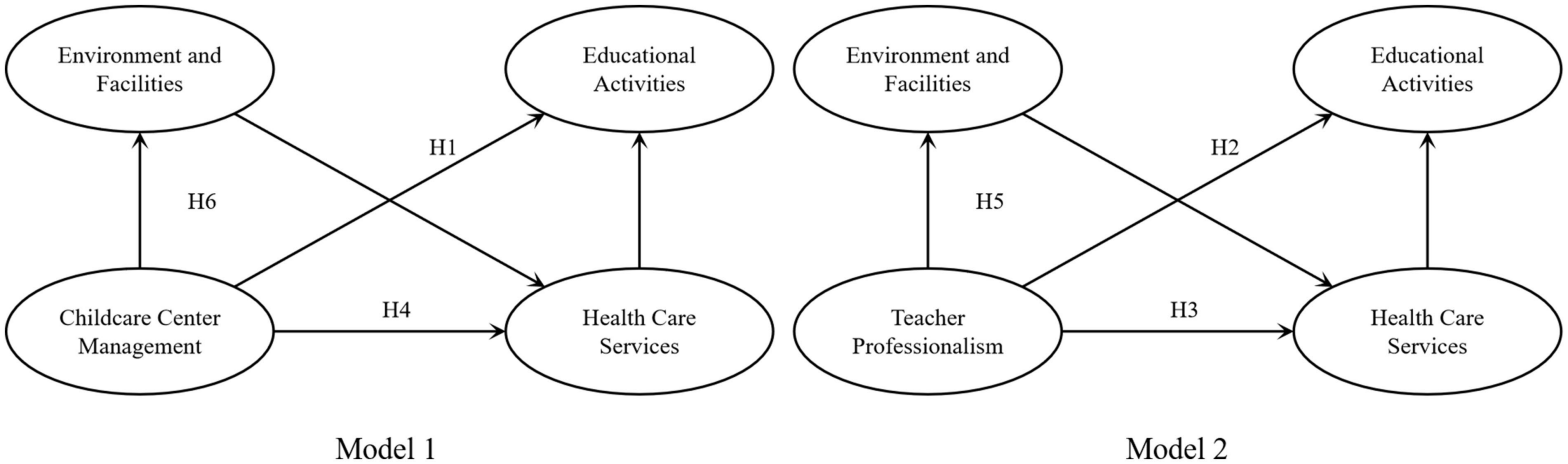
Theoretical model and research hypotheses.

Through GT analysis, the factors influencing childcare demand were divided into five core categories: CCM, TP, EF, HCS, and EA. To verify the scientific validity and reasonableness of the theoretical model, we proposed research hypotheses, designed questionnaires, obtained data, and analyzed the data using SPSS 29.0 to verify the research hypotheses. Because two independent variables point to the same mediator variable in the theoretical model, we split the model into two (Models 1 and 2) to facilitate subsequent data analysis (Figure 3). Among them, Model 1 has CCM as the independent variable, and Model 2 has TP as the independent variable. Based on the theoretical model derived from the analysis of GT and combining existing theories and research, we propose the following six research hypotheses:

*Hypothesis 1* (H1): TP positively affects EA.

*Hypothesis 1* (H2): CCM positively affects EA.

*Hypothesis 3* (H3): CCM affects EA through the HCS.

*Hypothesis 4* (H4): TP affects EA through the HCS.

*Hypothesis 5* (H5): CCM affects HCS through EF, which in turn affects EA.

*Hypothesis 6* (H6): TP affects HCS through EF, which in turn affects EA.

### 4.2 Questionnaire design

First, we extensively analyzed relevant literature, policy documents, and results of GT to develop quality indicators for the “*Questionnaire on the Childcare Needs of Parents of Children Aged 0–3*.” Second, regarding indicator and content validity, we sought the opinions of 15 experts through three interviews using the Delphi process. Experts recognized the indicators and made suggestions. Based on feedback from experts, we deleted some items. Third, parents from cities in Western China were recruited for the pilot study through convenience and purposive sampling. They were asked to complete the survey online and provide feedback on the wording of the items or other issues. Their feedback was collected and studied, and the corresponding items were refined to improve clarity and readability. The final version included 58 items categorized into five dimensions.

The questionnaire consisted of two parts: (1) Demographic characteristics of the study sample. Primary demographic information included age, guardians’ identity, educational level, career, geographical area, family structure, and number of children. (2) Scale of childcare needs of parents with children aged 0–3 years old. The scale consists of 58 questions, such as “The environment around the institution is suitable” or “The atmosphere in the institution is comfortable and warm, and the environment is created by the characteristics of children’s physical and mental development and attracts children.” The evaluation covered five dimensions: CCM, TP, EF, HCS, and EA. The scale was a 5-point Likert scale, with “1–5” corresponding to “strongly disagree, disagree, generally, agree, and strongly agree,” respectively. Ethical procedures were followed throughout this study. Parents were informed of the purpose of the survey at the beginning of the questionnaire and voluntarily agreed to participate in the test.

A total of 270 participants participated in the pretest to verify the reliability and validity of the questionnaire, and 238 valid questionnaires were returned. Subsequently, the reliability and validity of the questionnaire were tested using the SPSS 29.0. The data showed Cronbach’s alpha and KMO (Kaiser–Meyer–Olkin) for CCM, TP, EF, HCS, and EA. Dimensions. The overall Cronbach’s alpha and KMO for each pretest dimension were more than 0.9 (as shown in Table 5), indicating the questionnaire’s reliability and validity (57, 58). Therefore, the questionnaire was used as the main instrument for quantitative analysis in this study.

**Table 5:** Results of pretest reliability analysis.

	Cronbach’s alpha	KMO
CCM	0.973	0.929
TP	0.992	0.951
EF	0.973	0.938
HCS	0.980	0.954
EA	0.983	0.936
Overall	0.996	0.981

### 4.3 Participants

The questionnaire was released through the “Questionnaire Star” survey website. A stratified cluster sampling approach was adopted to recruit kindergarten teachers for the study. Previous studies have shown that highly educated young parents from core and Main families and with occupations in general management and technical staff are more likely to demand childcare services (8, 59, 60). In addition, scholars have pointed out that existing research has focused on the childcare needs of urban parents, while insufficient attention has been paid to the needs of childcare services in county towns and rural areas, where childcare services are still at a relatively late stage of development (61). Further study should be conducted on the childcare needs of parents in counties and rural areas to promote the balanced development of childcare services (62). To capture the perspectives of the main demand groups for childcare services, we sampled residents who were ready to have children and childcare and were already involved in childcare services in the Ili, Hotan, Urumqi, and Shihezi regions. We implemented purposive sampling by contacting respondents in pairs with the help of childcare center staff, maternity liaisons, early childhood education staff, and parents already involved in childcare services. A total of 530 questionnaires were collected. After excluding invalid questionnaires, 516 were retained with a validity rate of 97.4%.

Despite the relative youth in the sample, each decade of life is well-represented (63). The age of the interviewers was divided into three categories: less than 30 years (43.8%), 30–39 years old (44.4%), and more than 40 years (11.8%). According to Chen (64), the educational level of the participants was divided into six categories: junior school and lower (26.6%), senior high or vocational school (12.8%), some college (19.4%), and college degree and above (41.3%). According to Lin and Bian (65), respondents’ careers can be divided into five categories: temporary workers, unemployed, unemployable, unskilled, and agricultural workers (22.9%); manual workers (commercial service workers), self-employed, skilled workers, and workers of the same level (18.4%); general management versus general professional and technical staff (40.5%); middle management and mid-level professional and technical staff; assistant professionals (10.9%); senior professional executives (managers) and professional technicians; and professional supervisors (party and government leaders) (7.4%). According to the “Administrative Divisions of the People’s Republic of China (66),” the geographical areas of the sample covered rural areas (23.6%), townships (14.0%), countries (47.1%), and cities (15.3%). According to Zhou (67), the family structure includes the core family (parents and unmarried children) (50.6%), the main family (three generations of grandparents, parents, and their children) (21.9%), single-parent families (5.6%), intergenerational families (children living with grandparents) (9.7%), and others (12.2%). Participants’ demographic information is shown in Table 6.

**Table 6:** Summary of demographic characteristics of the study sample.

Name	Option	Frequency	Percentage (%)	Accumulative perception (%)
Age	Less than 30 years old	226	43.8	43.8
	30–39 years old	229	44.4	88.2
	More than 40 years old	61	11.8	100.0
Educational level	Junior school and lower	137	26.6	26.6
	Senior high or vocational school	66	12.8	39.4
	Some college	100	19.4	58.8
	College degree and above	213	41.3	100.0
Careers	Temporary workers, unemployed, unemployable, unskilled, and agricultural workers	118	22.9	22.9
	Manual workers (commercial service workers), self-employed, skilled workers, and workers of the same level	95	18.4	41.3
	General management versus general professional and technical, service staff	209	40.5	81.8
	Middle management and mid-level professional and technical staff, assistant professionals	56	10.9	92.6
	Senior professional executives (managers) and professional technicians, professional supervisors (party and government leaders)	38	7.4	100.0
Geographical area	Rural	122	23.6	23.6
	Township	72	14.0	37.6
	Country	243	47.1	84.7
	City	79	15.3	100.0
Family structure	Core family (parents and unmarried children)	261	50.6	50.6
	Main family (three generations of grandparents, parents, and their children)	113	21.9	72.5
	Single-parent family	29	5.6	78.1
	Intergenerational families (children living with grandparents)	50	9.7	87.8
	Others	63	12.2	100.0
Total		516	100	100.0

### 4.4 Data analysis and results

#### 4.4.1 Reliability and validity

To assess the adequacy of the samples, we used SPSS 29.0 to test the questionnaire’s Cronbach’s alpha and KMO values. The data show that Cronbach’s alpha was 0.996, greater than 0.9 (57). The KMO value was 0.981, which is greater than 0.9. Bartlett’s test of sphericity reached a significance level (*p* < 0.001) (58). The results indicate that the sample size was adequate. Next, we used AMOS 26.0 to examine the structural validity of the measurements.

As suggested by Kaiser and Hair (68, 69), before the reliability and validity of the construct can be checked, items with Cronbach’s alpha below 0.7 or loadings below 0.5 need to be removed. As shown in Table 7, Cronbach’s alpha for CCM, TP, EF, HCS, and EA were all higher than 0.7, and all the loading coefficients were higher than 0.5; therefore, we did not need to delete any items. Next, we examine composite reliability (CR) and average variance extracted (AVE). The result suggested that CR for all constructs ranged from 0.978 to 0.993, which is higher than 0.7 (69). The AVE for all constructs ranged from 0.787 to 0.911, higher than 0.5 (70).

**Table 7:** Reliability, discriminant validity.

Latent variable	Cronbach’s alpha	Loading	CR	AVE
CCM	0.982	0.939, 0.918, 0.959, 0.959, 0.942, 0.904, 0.959	0.982	0.884
TP	0.993	0.924, 0.964, 0.966, 0.965, 0.940, 0.962, 0.965, 0.959, 0.954, 0.975, 0.945, 0.939, 0.946	0.993	0.911
EF	0.980	0.800, 0.862, 0.898, 0.700, 0.908, 0.916, 0.925, 0.906, 0.895, 0.947, 0.931, 0.926	0.978	0.787
HCS	0.988	0.909, 0.936, 0.915, 0.898, 0.921, 0.918, 0.913, 0.896, 0.938, 0.948, 0.942, 0.923, 0.898, 0.907, 0.916	0.988	0.844
EA	0.988	0.904, 0.916, 0.934, 0.913, 0.931, 0.941, 0.963, 0.958, 0.959, 0.959, 0.923	0.987	0.877

#### 4.4.2 Descriptive statistics and correlation

This study examined the characteristics of five latent variables. As shown in Table 8, the coverage values of all variables were greater than 4. This indicates that parents have a high demand for all five variables. Parents’ childcare needs were, from largest to smallest, TP (*M* = 4.488, SD = 0.944), HCS (*M* = 4.477, SD = 0.922), EF (*M* = 4.448, SD = 0.935), CCM (*M* = 4.440, SD = 0.961), and EA (*M* = 4.436, SD = 0.960). At the same time, the absolute value of the kurtosis of each dimension is less than 8, and the absolute value of the skewness is less than 3, which indicates that the data, although not absolutely normal, is basically acceptable as a normal distribution (71). In addition, based on the Pearson correlation analysis, it can be seen that the correlation coefficients between the factors were higher than 0.8, and all showed positive and significant correlations (*p* < 0.001) (72).

**Table 8:** Descriptive statistics of correlation data.

Latent variable	Mean	SD	Skewness	Kurtosis	CCM	TP	EF	HCS	EA
CCM	4.440	0.961	−1.945	3.329	1				
TP	4.488	0.944	−2.058	3.790	0.937***	1			
EF	4.448	0.935	−1.928	3.160	0.874***	0.887***	1		
HCS	4.477	0.922	−2.088	4.043	0.929***	0.949***	0.909***	1	
EA	4.436	0.960	−1.919	3.205	0.910***	0.945***	0.901***	0.952***	1

*p < 0.05, **p < 0.01, ***p < 0.001.

### 4.5 Hypothesis testing

Previous studies have pointed out that the Bootstrap method is helpful in reducing the adverse effects of multicollinearity and improving model robustness (73). Also, the Bootstrap method requires a sample size of at least 50 cases, and the sample size in this study met this criterion (74). Therefore, the study used Bootstrap methodology to examine the promotional effect of EF on HCS and its chain mediating role between CCM with EA and TP with EA. In reference to the majority of scholars, we embedded the PROCESS program into SPSS, selected 95% confidence intervals, and repeated the sampling 5,000 times. In addition, it was tested to provide relatively stable confidence intervals at 5,000 samples. Previous studies have shown that parental age, education level, occupation, location, family structure, and number of children significantly influence parental demand for childcare (28–33). This study included the above variables as control variables in all data analysis models. Table 9 presents the model fit indicators and regression coefficients of the mediating role model. According to the regression results, the *p*-values were less than 0.001, and the 95% confidence intervals did not contain 0, indicating that the model was well-fitted. Table 10 reports the results of the mediating effect sizes and percentages.

**Table 9:** Variable regression results.

Outcome variable	Predictive variable	*R^2^*	*F*	*β*	SE	*t*	LLCI	ULCI
EF	CCM	0.769	241.067	0.862***	0.022	37.591	0.795	0.882
HCS	CCM	0.904	596.172	0.570***	0.028	19.861	0.493	0.601
	EF			0.418***	0.028	14.604	0.357	0.468
EA	CCM	0.919	638.630	0.142***	0.035	4.034	0.073	0.211
	EF			0.181***	0.032	5.783	0.123	0.249
	HCS			0.656***	0.042	16.080	0.599	0.766
EA	CCM (Total effect)	0.830	353.728	0.908***	0.019	46.611	0.869	0.908
EF	TP	0.793	278.847	0.869***	0.021	40.927	0.819	0.901
HCS	TP	0.922	751.155	0.666***	0.026	24.623	0.598	0.702
	EF			0.319***	0.027	11.692	0.262	0.367
EA	TP	0.930	743.301	0.371***	0.039	9.744	0.301	0.454
	EF			0.152***	0.030	5.184	0.097	0.215
	HCS			0.461***	0.044	10.906	0.393	0.566
EA	TP (Total effect)	0.895	617.011	0.938***	0.015	61.872	0.923	0.983

*p < 0.05, **p < 0.01, ***p < 0.001.

**Table 10:** Bootstrap test results for mediating effects.

Effect	Effect value	Boot SE	Boot LLCI	Boot ULCI	The ratio of indirect to total effect
CCM-EA					
Total effect	0.908	0.019	0.869	0.908	
Direct effect	0.142	0.035	0.073	0.211	
Total indirect effect	0.766	0.063	0.630	0.873	84.36%
Ind1: CCM-EF-EA	0.156	0.088	−0.003	0.336	17.18%
Ind2: CCM-HCS-EA	0.374	0.060	0.254	0.491	41.19%
Ind3: CCM-EF-HCS-EA	0.236	0.068	0.120	0.387	25.99%
TP-EA					
Total effect	0.938	0.015	0.923	0.983	
Direct effect	0.371	0.039	0.301	0.454	
Total indirect effect	0.566	0.075	0.417	0.711	60.34%
Ind1: TP-EF-EA	0.132	0.093	−0.042	0.318	14.07%
Ind2: TP-HCS-EA	0.307	0.070	0.165	0.438	32.73%
Ind3: TP-EF-HCS-EA	0.128	0.041	0.060	0.221	13.65%

As shown in Table 9, the positive direct effect of TP on EA was significant (*β* = 0.371, *p* < 0.001), and the positive direct impact of CCM on EA was also significant (*β* = 0.142, *p* < 0.001). The stability of the results was further validated by the fact that both sets of results showed 95% confidence intervals that did not contain 0. The empirical results verified H1 and H2. Similarly, CCM significantly affected HCS (*β* = 0.870, *p* < 0.001), and HCS significantly affected EA (*β* = 0.656, *p* < 0.001). The confidence intervals for the two datasets do not include 0, which suggests that HCS acts as a mediating variable between CCM and EA. The empirical results support H3. TP significantly affected HCS (*β* = 0.666, *p* < 0.001), and HCS significantly affected EA (*β* = 0.461, *p* < 0.001). The confidence intervals for the two datasets do not include 0, suggesting that HCS is a mediating variable between TP and EA. The empirical results support H4.

Table 10 shows that the chain-mediated effect size of EF and HCS between CCM and EA is 0.236, with a confidence interval of [0.120, 0.387], excluding 0. The chain-mediated effects were also significant. Thus, H5 was validated using the data results. EF and HCS had a chain-mediated effect size of 0.128 between TP and EA, with a confidence interval of [0.060, 0.221], excluding 0. The chain-mediated effects were also significant. The empirical results verified that H6.

**Figure 4:**
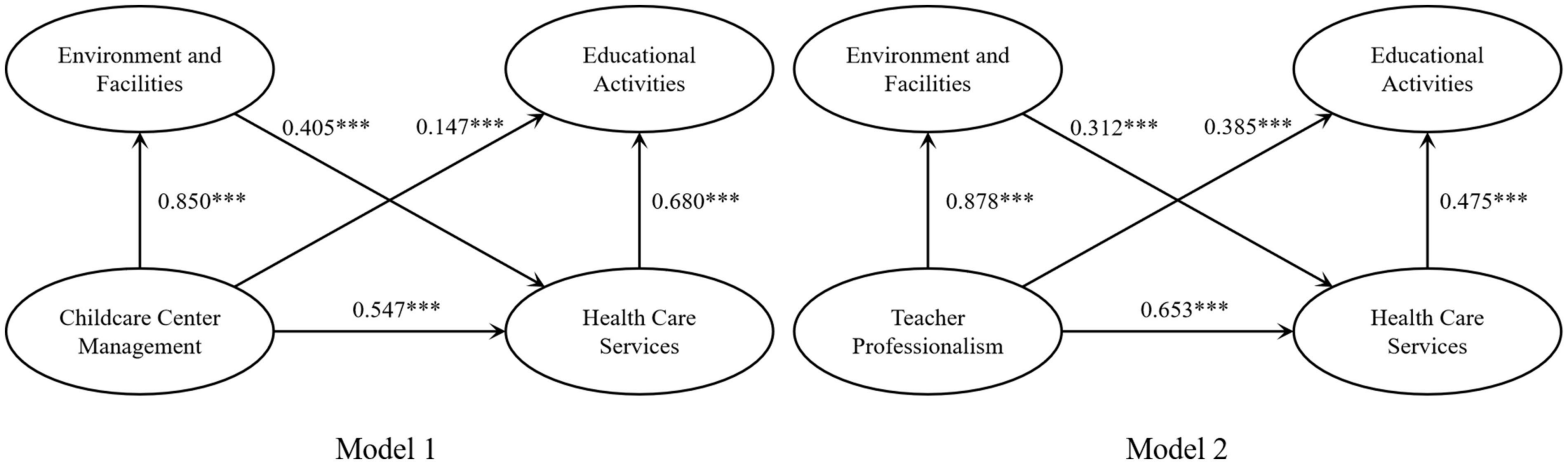
Theoretical model validation results. *p < 0.05, **p < 0.01, ***p < 0.001.

The hypothesized theoretical model focuses on whether the effects between variables hold, namely, whether TP and CCM affect EA, whether the mediating impact of HCS is significant, and whether the chain-mediating effect of EH in promoting HCS is significant between TP with EA and CCM with EA. As shown in Figure 4, the path coefficients between all Model 1 and Model 2 variables are significant. Based on the results of the mediation effects analysis, it can be seen that both the theoretical model and the research hypotheses are valid.

## 5 Discussion

First, semi-structured interviews were conducted with a total of 63 stakeholders, including parents of children aged 0–3 years, childcare center managers, teachers, education specialists and community members. Second, three levels of coding of the interview data using GT were used to summarize the factors influencing the parenting needs of parents of 0–3-year-old children, including CCM, TP, EF, HCS, and EA. Third, we combined relevant theories to construct a theoretical model of the factors influencing parents’ childcare services for children aged 0–3 years old and formulated research hypotheses. Fourth, a questionnaire was used to obtain quantitative data and test the research hypotheses in conjunction with regression and mediation analyses. Finally, three main conclusions were drawn: (1) Correlation analysis showed a significant positive correlation between CCM, TP, EF, HCS, and EA. (2) Regression analyses showed that CCM and TP positively and directly influenced EA. (3) Analysis of the mediating roles showed that HCS mediated between CCM and EA, HCS mediated between TP and EA, EF and HCS chained between CCM and EA, and EF and HCS chained between TP and EA. Herein, we discuss the findings of this study.

### 5.1 Direct impact

This study had two findings regarding direct effects: CCM positively and directly affects EA. The CCM is an operating fundamental of childcare centers, which is essential for the quality of the center’s education. This means the higher the CCM quality, the better the EA quality. The results of this study are similar to those of a previous study. As stated in Weick’s occupational socialization theory (75), leaders and workers of childcare centers form many childcare practices and standards through consultation and communication, providing support for the teaching activity, facilitating communication, and co-constructing consensus among teachers, which boosts the quality of EA. Bayly et al. also noted that one factor affecting the quality of childcare programs may be the management of childcare facilities (76). Specifically, childcare centers are managed using a business model that meets the needs of employers and seeks to maximize shareholder profits rather than focusing on the needs of parents and children and the quality of early education (76, 77). In addition, Yang and Lim pointed out that if the instructional management system in childcare institutions is not reasonable, it will cause tremendous organizational and instructional pressure to achieve the vision of realizing distributed instructional leadership and improving the quality of education in childcare institutions (78).

Management should be used to enhance the quality of education in childcare centers and improve the quality of the process. Specifically, in the management philosophy, childcare centers should establish the concept of self-management and continuous improvement as an unchanging pursuit. For example, the National Association for the Education of Young Children (NAEYC) quality accreditation process begins with the self-reflection of all staff members (79). Regarding management content, childcare centers should focus on managing the quality of the process, such as strengthening the curricular leadership of directors and teachers and creating a good learning environment for children (80). Regarding administration, childcare centers should break the top-down single leadership paradigm and lead the change of leadership paradigm to form an atmosphere of goal sharing, mutual respect, wisdom integration, power and responsibility sharing, and democratic management in the childcare centers (81).

Second, TP positively and directly affects EA. TP is the basis of EA and plays a decisive role in the quality of education in childcare centers. This means the higher the TP quality, the better the EA quality. The study by Hunter et al. supports the results of this study. Hunter et al. found that teachers’ teaching skills and receptivity to intervention counseling can predict the quality of the childcare centers’ teaching methods (82). Other research has suggested that childcare teachers’ professional competence positively correlates with infants’ development, future academic achievement, and individual growth (83). Teachers are in close contact with young children, and with the support of professional concepts, knowledge, and skills, they carry out educational activities based on their understanding of the children, which improves the quality of education.

Teacher quality is the core and key to the quality of childcare, and improving the quality of the childcare process requires a higher level of childcare teachers (84). Based on this, the study concluded that the quality of childcare teachers can be improved in three ways: first, strengthening the training of talents in childcare services and enhancing their capacity to supply them. Support universities and colleges with conditions to develop relevant disciplines and specialties, such as infant and child development, health management, and infant and childcare (85). Second, the training of childcare professionals should be strengthened, and paths should be explored to upgrade “childcare workers” to “childcare masters” to improve caregivers’ comprehensive quality and professional skills (86). Third, access and evaluation systems for childcare teachers should be improved. International experience has shown that professional emotions, love, knowledge, and competence are emphasized in the qualifications for access to childcare teachers. Knowledge and competence criteria are also translated into the basis for assessment, certification, and auditing access to qualifications (87).

### 5.2 The mediating role of HCS

It was found that the HCS is an intermediary variable between CCM and EA. Improving the CCM level contributes to the HCS’s quality and enhances the EA’s quality. This result confirms the conclusions of existing studies that effective CCM is an essential factor in the quality of health care in childcare centers (81) and that health care is a powerful means of ensuring young children’s right to survival and development and an essential support for effective EA (88). Refined management often establishes a scientific safety and health system and a system of childcare services to ensure the safety of children’s lives and physical health (49) and to provide fundamental safeguards for educational activities. In contrast, the absence of a scientific management philosophy and standardized management system will lead to lower childcare center efficiency and HCS quality. Eventually, the operation of educational activities is affected.

The present study also found that HCS mediated the relationship between TP and EA. This means that the level of TP affects the quality of the HCS, which in turn affects EA. This mirrors the findings of Werner et al. (89), who noted that improving teacher professionalism is often effective in improving childcare quality, caregiver interaction skills, and child development. Therefore, as a crucial subject in infant and toddler education, teachers in childcare centers are essential guides and companions for infant and toddler development. The professionalism and competence of teachers play a crucial role in the healthy physical and mental growth of infants and toddlers, as well as in the development of their personalities (90).

This shows that HCS are an essential basis for improving the quality of education in childcare centers. We make the following recommendations to improve the quality of the following recommendations. First, we established the scientific concept of healthcare to provide ideological guidance for childcare centers. Teachers’ lack of knowledge about children’s healthcare can make it difficult for childcare centers to practice proper healthcare, leading to a disconnect between teaching and learning (91). Second, having a written policy can provide the organization with a framework for decision-making and allow employees to understand their roles and responsibilities (92). Therefore, relevant departments should introduce a system of professional healthcare workers as soon as possible, formulate professional qualification standards, and clarify job responsibilities (93). Dietary education planning, daylife dietary guidance for young children, and healthcare management can be taken as the responsibility of the healthcare teacher to build a specialized healthcare teacher team. Third, training on “healthcare knowledge and skills” has been organized for teachers and healthcare workers to enrich their knowledge and skills to provide scientific guidance for childcare centers (94).

### 5.3 The chain mediating role of EF and HCS

Two pathways have been identified that play a role in chain mediation. (1) EF and HCS are chain mediators between CCM and EA. (2) EF and HCS mediated TP and EA. Among them, EF and HCS are chain mediators between CCM and EA, which means that CCM influences EA through the combined effects of EF and HCS. The conclusions of this study support those of the previous studies. Previous studies (95, 96) have shown that managing childcare institutions affects the quality of the spatial environment. By contrast, the quality of the spatial environment affects children’s physical health, which in turn affects the quality of education in early childhood institutions. The management of childcare centers includes choice of location, spatial environment design, and facilities provision. The outdoor and indoor environments of childcare centers are essential for children’s physical and mental development. When the health and safety of children is compromised, it is difficult for educational activities to take place.

EF and HCS are chain mediators between TP and EA, indicating that TP influences EA through the interaction between EF and HCS. This finding is similar to the philosophy of Luo and Liu (97), who believe that teachers are central to improving and ensuring the quality of childcare organizations. When teachers use appropriate facilities and materials to engage infants and toddlers in activities and provide them with learning opportunities, they can develop, and educational activities can be meaningful. Guided by professional philosophy and knowledge, teachers provide children with appropriate learning environments, facilities, and materials. The learning environment affects how a child thinks, feels, and behaves. This is critical for developing a child’s self-concept and identity (98). Specifically, teachers consider the value and function of different environmental systems when constructing the curriculum and emphasize the organic linkages between the diverse elements of the environment to enhance the foundational and sustainable nature of young children’s development (99).

Therefore, improving the quality of EF is essential to improve the quality of HCS. The study concludes that the quality of EF can be enhanced in several ways. First, childcare providers should focus on increasing awareness and knowledge of environmental health issues (100). Second, increasing the natural elements of childcare centers promotes physical activity and other positive health effects on children (101). For example, adding appropriate natural features such as trees, flowers, small animals, sandboxes, and pools provides opportunities for young children to get close to nature. Finally, some scholars suggest that the environmental design of childcare institutions should be based on a two-way perspective for young children (102), considering not only their physiological structures but also their psychological functions. Environmental design should stimulate young children’s motivation and curiosity and gradually cultivate lifelong learning habits.

### 5.4 Research contribution and limitation

As the mixed research method realizes the complementary roles of qualitative and quantitative methods, it allows for a more in-depth and comprehensive exploration of the research problem (103). Therefore, this study adopts a mixed-method approach to understand the factors influencing the demand for parental childcare for children aged 0–3 years in China and its operating mechanism.

Relative to previous studies, this study makes several contributions: (1) The study mainly focuses on the parents of 0–3-year-old children as the research object, from the micro level of parents to understand their demand for childcare service influencing factors and their role in the mechanism, which is conducive to supplementing the theoretical content of 0–3-year-old infants and toddlers in childcare services, and provides a reference for future theoretical research. (2) Exploring the factors influencing parents’ demand for childcare and the relationship between those factors is conducive to understanding the current situation of parental childcare to accurately provide childcare services, balance the contradictions between women’s families and their work, and revitalize the willingness of the appropriate age group to have children. (3) This study used a mixed research method to explore the factors influencing parents’ childcare needs for children aged 0–3 years and their mechanisms of action, thus providing a new research paradigm for studying childcare centers and parents’ childcare needs in other countries and regions.

Despite the many contributions of this study, it still has some shortcomings: (1) Whether there are reverse paths between the factors in the theoretical model has to be verified and can be explored in a more profound step in the future. (2) This study was cross-sectional and did not track parental childcare needs; however, parental needs for childcare may change over time and as children grow. Therefore, a tracking study of parental childcare needs should be conducted at different points to provide more valuable references for childcare providers to meet parental needs. (3) Since we only sampled some districts, the sampling method and sample size may have introduced a slight bias in the data results, which potentially limits the generalizability of the findings. We acknowledge that these results may be more applicable to the specific region or country studied, given the influence of local cultural attitudes, practices, politics, and organizational structures. Future studies will refine the sampling strategy to ensure a representative and randomized sample, thereby mitigating issues related to uneven sample sizes and enhancing the potential for broader applicability.

In summary, a mixed-methods approach was used to explore the factors influencing childcare demand by parents of children aged 0–3 years and their operating mechanisms. First, through semi-structured interviews and GT, the study summarized the factors influencing the childcare needs of parents of 0–3-year-old children, including CCM, TP, EF, HCS, and EA. Second, the theoretical model combines the relevant theories and proposes six hypotheses. Third, questionnaires were used to obtain quantitative data and to test the research hypotheses in conjunction with regression and mediation analyses. Finally, three main conclusions are drawn. (1) Correlation analysis showed significant positive correlations among CCM, TP, EF, HCS, and EA. (2) Regression analysis revealed that CCM and TP had positive and direct effects on EA, respectively. (3) Mediating role analysis showed that HCS mediated between CCM with EA and TP with EA, EF, and HCS acting as chain mediators between CCM with EA and TP with EA. This study provides theoretical references and a practical basis for solving the imbalance between the supply and demand for childcare, promoting the effective development of infant and childcare services, and improving the quality of childcare services.

## Data Availability

The raw data supporting the conclusions of this article will be made available by the authors without undue reservation.
